# Acknowledgement to Reviewers of *Antioxidants* in 2016

**DOI:** 10.3390/antiox6010004

**Published:** 2017-01-11

**Authors:** Antioxidants Editorial Office

**Affiliations:** MDPI AG, St. Alban-Anlage 66, 4052 Basel, Switzerland; antioxidants@mdpi.com

The editors of *Antioxidants* would like to express their sincere gratitude to the following reviewers for assessing manuscripts in 2016. 

We greatly appreciate the contribution of expert reviewers, which is crucial to the journal’s editorial process. We aim to recognize reviewer contributions through several mechanisms, of which the annual publication of reviewer names is one. Reviewers receive a voucher entitling them to a discount on their next MDPI publication and can download a certificate of recognition directly from our submission system. Additionally, reviewers can sign up to the service Publons (https://publons.com) to receive recognition. Of course, in these initiatives we are careful not to compromise reviewer confidentiality. Many reviewers see their work as a voluntary and often unseen part of their role as researchers. We are grateful to the time reviewers donate to our journals and the contribution they make. 

If you are interested in becoming a reviewer for *Antioxidants*, see the link at the bottom of the webpage http://www.mdpi.com/reviewers.

The following reviewed for *Antioxidants* in 2016:
Abourashed, EhabGrilli, EsterMouithys-Mickalad, AngeAmorati, RiccardoGrimm, Marcus O.W.Mulinacci, NadiaAmorós, AsunciónGupton, B. FrankMusgrave, IanArihara, KeizoHaider, LukasNaito, YujiAugé, NathalieHarper, JamesNauser, ThomasAzadfar, MohammadaliHarris, Cory S.Nazzaro, FilomenaBallmann, ChristopherHeckman, KarinO’Callaghan, YvonneBanati, Richard B.Hegde, Muralidhar L.Olson, KennethBarreiro, Sonia LosadaHellens, RogerPanzella, LuciaBarry-Ryan, CatherineHernández, FranciscaPaoli, PaoloBarsberg, Søren TalbroIchinose, KojiPappas, ChristosBekhit, Alaa El-Din AhmedIrato, PaolaPardini, RonBellés, José MaríaIwaoka, MichioParola, MaurizioBendich, AdrianneJones, MerielPereira, Olívia R.Birch, JohnKabil, OmerPereira, VandaBlanton, CynthiaKahlert, StefanPetrucci, RitaBobe, GerdKarokoti, AjayPintore, Giorgio Antonio MarioBosch, ChristineKatiyar, Santosh K.Pognonec, PhilippeBreckwoldt, MichaelKay, Colin D.Poojary, MaheshaBurton-Freeman, BrittKelley, Eric E.Proestos, CharalamposCaccetta, RimaKing, CharlesPutnik, PredragCampbell, Fiona M.Klims-Zacas, DorothyReddy, P. HemachandraCampos, NarcisoKnupp, GerdResconi, Virginia CeliaCankaya, IremKolb, AndreasRicelli, AlessandraCarvalho, PauloKostyuk, Vladimir A.Roderfeld, MartinCavazza, AntonellaKumazawa, ShigenoriRodriguez-Mateos, Ana M.Chen, TongKurdowska, Anna K.Rubis, BlazejCherkaoui-Malki, MustaphaLa Favor, Justin D.Russo, NinoChiang, Wen-DeeLaight, DavidRybalka, EmmaChiva-Blanch, GemmaLalas, StavrosSaidu, NathanielChohan, MagaliLandete, José MariaSchrag, MatthewCocero, Maria JoseLee, JunsooSchuck, AlyssaConte, AngelaLehtonen, MariSendón, RaquelCulotta, Valeria C.Leibowitz, AvshalomSimes, DinaDel Bò, CristianLizard, GerardStamatopoulos, KonstantinosDonno, DarioLloret, AnaStewart, AmandaDurazzo, AlessandraLohning, AnnaStoner, Gary D.Eckl, PeterManocha, GunjanStuart, JeffFabbrocini, GabriellaMaret, WolfgangSurai, PeterFernandez, Maria LuzMarkopoulou, OlgaSvoronos, ParisFilipovic, Milos R.Martínez, SidoniaTavazzi, BarbaraFracessetti, DanielaMatute, CarlosTayebati, Seyed KhosrowFrancesca, BonominiMcAnulty, LisaTergaonkar, VinayGaffo, Angelo L.McDougall, GordonTeschke, RolfGail, WelsbyMcRae, Jacqui M.Ushio-Fukai, MasukoGaleano-Díaz, TeresaMedbury, HeatherVarzakas, TheodorosGalli, FrancescoMelkani, GirishVasin, Mikhail V.Gan, RenyouMellor, Duane D.Vorsa, NicholiGandhi, SoniaMelnik, Bodo C.Walters, DianneGarten, RyanMinbiole, Kevin P.C.Wang, XinkunGaucher, CarolineMitakou, SofiaWilliams, Carey A.Georgakilas, Alexandros G.Miura, KyokoWu, Jian-PingGieseg, StevenMøller, Ian MaxWu, YingGirgih, Abraham T.Moon, Gi-seongYazlovitskaya, Eugenia M.Giuffrè, Angelo MariaMoon, Sun HeeZadra, ClaudiaGreenspan, PhillipMoorhead, Greg
Grillari, JohannesMoreau, Regis


